# *Francisella* IglG protein and the DUF4280 proteins: PAAR-like proteins in non-canonical Type VI secretion systems?

**DOI:** 10.15698/mic2016.11.543

**Published:** 2016-10-29

**Authors:** Claire Lays, Eric Tannier, Thomas Henry

**Affiliations:** 1 CIRI, Centre International de Recherche en Infectiologie, Inserm, U1111, Université Claude Bernard Lyon 1, CNRS, UMR5308, École Normale Supérieure de Lyon, Univ Lyon, F-69007, LYON, France.; 2 LabEX Ecofect, Eco-evolutionary dynamics of infectious diseases, Lyon, France.; 3 INRIA Rhône-Alpes, 655 av de l’Europe, F-38334 Montbonnot, France, LBBE, UMR5558, Université Claude Bernard Lyon 1, Univ Lyon, F-69007, Lyon, France.

**Keywords:** Francisella, DUF4280, PAAR, T6SS, PF14107, phage, type VI secretion system

## Abstract

Type VI secretion systems (T6SS) are bacterial molecular machines translocating effector proteins into target cells. T6SS are widely present in Gram-negative bacteria where they predominantly act to kill neighboring bacteria. This secretion system is reminiscent of the tail of contractile bacteriophages and consists of a contractile sheath anchored in the bacterial envelope and an inner tube made of stacks of the Hcp protein. The Hcp tube is capped with a VgrG trimer and a spike protein termed PAAR, which acts as the membrane-puncturing device. *Francisella tularensis*, the agent of tularemia, is an intracellular bacterium replicating within the host cytosol. Upon entry into the host cell, *F. tularensis* rapidly lyses the host vacuolar membrane to reach the host cytosol. This escape is dependent on the *Francisella* Pathogenicity Island (FPI), which is encoding an atypical T6SS. Among the 17 proteins encoded by the FPI, most of them required for virulence, eight have some homology to canonical T6SS proteins. We recently identified the function of one protein of unknown function encoded within the FPI, IglG. By three-dimensional modelling and following validation by different techniques, we found that IglG adopts a fold resembling the one of PAAR proteins. Importantly, IglG features a domain of unknown function DUF4280, present in numerous bacterial species. We thus propose to rename this domain of unknown function, PAAR-like domain, and discuss here the characteristics of this domain and its distribution in both Gram-negative and Gram-positive bacteria.

PAAR proteins are characterized by 3 proline-alanine-alanine-arginine motifs and by the ability to coordinate a zinc ion by 3 histidine and one cysteine residues. This metal binding is thought to stabilize their 3D structure. PAAR proteins are localized at the tip of T6SS where they interact with the trimeric VgrG protein. In our recent study, we identified that IglG and DUF4280 proteins are predicted to adopt a similar fold as PAAR proteins. IglG and DUF4280 proteins are characterized by 4 highly conserved cysteine residues. These 4 cysteine residues are essential for IglG function in the T6SS, for virulence in a mouse model of tularemia and were demonstrated to contribute to the coordination of a metal ion (either zinc or iron). The predicted structure of IglG and DUF4280 consensus sequences and their ability to bind metal led us to propose that IglG and DUF4280 proteins are bona fide PAAR-like proteins.

Present in numerous proteins of unknown function, the DUF4280 domain (PF14107) is a domain of about 100 amino acids with 4 conserved cysteine residues. It is found in more than 250 bacterial species, including both Gram-positive and Gram-negative bacteria. A phylogenetic analysis demonstrates that PAAR-like proteins form a clade distinct from typical PAAR proteins and from their bacteriophage homologues (Gp5.4 proteins), suggesting an ancient divergence (Figure 1). The presence of PAAR-like proteins in Gram-positive bacteria (highlighted in blue in figure 1) is remarkable, given the fact that T6SS have been described so far only in Gram-negative bacteria. DUF4280 proteins from Gram-positive species appear as relatively grouped in the phylogenetic tree. Yet, their sequences do not diverge much from Gram-negative DUF4280 sequences, suggesting a common history (and a possible common function) for the PAAR-like proteins in Gram-positive and Gram-negative bacteria. This raises the fascinating possibility that Gram-positive bacteria may possess functional phage-like translocation system sharing homology with Gram-negative T6SS.

**Figure 1:**
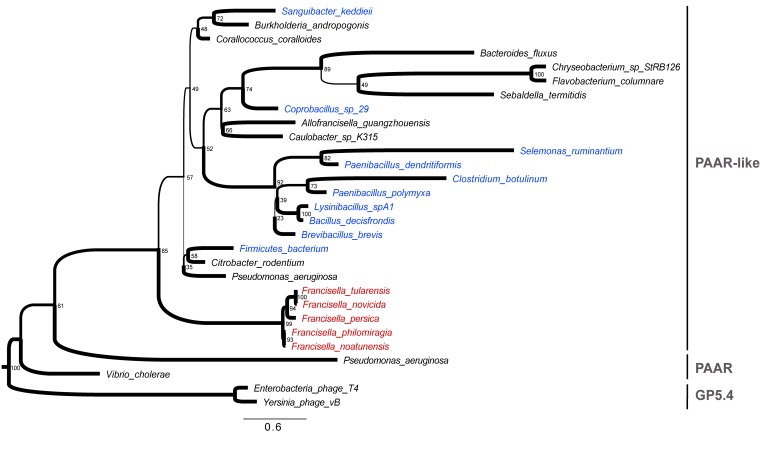
FIGURE 1: Phylogenetic relationships of PAAR-like, PAAR and GP5.4 bacteriophages sequences. The tree was built using IQtree with 1000 bootstrap replicates on a set of sequences retrieved from PFAM (PF14107). 2 PAAR and 2 GP5.4 sequences were added for comparison. All sequences and methodological details are available upon request. The tree was rooted with the phage sequences. Tree branch widths were set according to their bootsrap values (see number at the nodes). The scale bar represents the average number of amino acid replacements per site.

In the PAAR-like phylogeny, *Francisella* species (highlighted in red) appear as phylogenetic outliers in line with previous phylogenetic analyses performed by Bingle and collaborators using TssB/C (IglA/B) sequences. The divergence of *Francisella* PAAR-like protein and T6SS might be linked to its unique ability to target the eukaryotic phagosomal membrane. With the characterization of IglG as a PAAR-like protein, the three *Francisella* homologues of the canonical T6SS inner tube proteins are now identified. IglC, VgrG and IglG have structural homology with the Hcp, VgrG and PAAR proteins. Yet, experimental evidence is still lacking to demonstrate that these proteins function together to form the *Francisella* T6SS inner tube. Surprisingly, while IglG is fully required for the intracellular life cycle of *F. novicida*, it is only delaying the intracellular life cycle of a highly virulent *F. tularensis* strain. This result, although not fully understood, suggests that in some bacteria, T6SS might have a residual activity in absence of PAAR/PAAR-like proteins. Importantly, deletion of *iglG* renders all the tested strains completely avirulent in a mouse model of tularemia, indicating that a fully functional T6SS with a PAAR-like protein is required *in vivo* for virulence. As of today, it is still unknown whether the FPI encodes a T6SS effector, which would be directly responsible for the vacuole lysis. Importantly, similarly to some VgrG proteins, some PAAR and PAAR-like proteins feature N- or C-terminal extension with predicted enzymatic domains (e.g. endonuclease or lipase) suggesting that PAAR and PAAR-like domain could act as cargo to deliver effectors into the target cell. IglG possesses a short amphiphilic α-helical extension in the N-terminus of the PAAR-like domain. This IglG domain binds IglF, another FPI protein required for virulence. The role of IglF remains to be established but based on its interaction with IglG, we speculate that IglF might be translocated into the host cell together with IglG and act as an effector. Deciphering the mechanism controlling vacuole lysis in relation to the activity of this atypical T6SS is without doubt one of the most exciting challenge in the *Francisella* field.

